# A new agent developed by biotransformation of polyphyllin VII inhibits chemoresistance in breast cancer

**DOI:** 10.18632/oncotarget.6674

**Published:** 2015-12-19

**Authors:** Dong-Xu He, Guo-Hong Li, Xiao-Ting Gu, Liang Zhang, Ai-Qin Mao, Juan Wei, De-Quan Liu, Gui-Yang Shi, Xin Ma

**Affiliations:** ^1^ School of Pharmaceutical Sciences National Engineering, and Laboratory for Cereal Fermentation Technology, Jiangnan University, Wuxi 214122, China; ^2^ Laboratory for Conservation and Utilization of Bio-Resources, and Key Laboratory for Microbial Resources of The Ministry of Education, Yunnan University, Kunming 650091, China; ^3^ Department of Breast Surgery, The Third Affiliated Hospital, Kunming Medical University, Kunming 650031, China

**Keywords:** biotransformation, endophyte, polyphyllin VII, cancer, chemoresistance

## Abstract

Biotransformation by the endophytes of certain plants changes various compounds, and this ‘green’ chemistry becomes increasingly important for finding new products with pharmacological activity. In this study, polyphyllin VII (PPL7) was biotransformed by endophytes from the medicinal plant *Paris polyphylla* Smith, var. *yunnanensis*. This produced a new compound, ZH-2, with pharmacological activity *in vitro* and *in vivo*. ZH-2 was more potent than PPL7 in selectively killing more chemoresistant than chemosensitive breast cancer cells. ZH-2 also re-sensitized chemoresistant breast cancer cells, as evidenced by the improved anti-cancer activity of commonly-used chemotherapeutic agent *in vitro*, *in vivo*, and in clinical samples. This anti-chemoresistance effect of ZH-2 was associated with inhibiting the epithelial-mesenchymal transition (EMT) pathway. Taken together, our findings are the first one to link biotransformation with a biomedicine. The results provide insights into developing new pharmacologically-active agents *via* biotransformation by endophytes.

## INTRODUCTION

Biotransformation is a process by which biological systems such as enzymes and whole-cell metabolism produce chemical changes in various compounds [1;2]. Unlike compounds produced by chemical methods, biotransformation shows high reaction-, enantiomer-, and region-specificity because of the enzyme specificity in biological systems. Furthermore, the great variety of versatile enzymes can simplify the process of chemically altering complex molecules in milder and more environmentally-friendly conditions than standard chemical catalysts [3]. Consequently, in the last ten years, biotransformation has become an increasingly important tool in the development of pharmacologically-active agents [4;5] such as compound that are antiviral [6], reduce somatic fat [7;8], or regulate blood pressure [9], among others [10].

Microorganisms are an important source of catalysts for biotransformation because their enzyme systems are capable for converting unfamiliar substrates. The typical procedure of microbial biotransformation is carried out in a microbial culture system with added substrate. Then, one or more microbial enzymes recognize the substrate and carry out reactions to change its structure. Endophytes are one of the microorganisms used to biotransform active compounds from their host plants [10]. Endophytes are bacterial or fungal microorganisms that live symbiotically with their host plant. During co-evolution with the host plants, they have developed unique enzymes and metabolic systems to perform acylation, oxidation, reduction, hydrolysis, and nitration of active compounds in their hosts [4]. Therefore, their diverse biotransformation systems show great potential for transforming a wide range of molecules for botany, physiology, and pharmacology. For example, ginsenosides from *Panax notoginseng* have been clinically shown to have various biological activities, and biotransforming the ginsenoside Re *via* endophytes of *P. notoginseng* generated new product, compound K [11], which is more pharmacologically active than Re.

*Paris polyphylla* Smith, var. *yunnanensis* (Franch) is an established herbal medicine in China for the treatment of fevers, headaches, burns, wounds, and, over the past decade, cancers [12]. Polyphyllins and diosgenins are major saponins of *P. polyphylla*, and they show a wide range of biological actions, including anti-tumor activity. For example, polyphyllin D inhibits growth of the breast cancer cells MCF-7 and MDA-MB-231, as well as decreasing the volume of xenografts formed by these cells [13]. Also, saponins from *P. polyphylla* inhibit growth in the liver cancer cell line HepG2 [14], decrease proliferation in the cervical adenocarcinoma cell line HeLa [15], and display other biological activities including anti-leishmaniasis [16] and immuno-stimulating [17] actions.

We designed this study to investigate the biotransformation of polyphyllins by endophytes from *P. polyphylla*, and assess the pharmacological activity of the resulting products. The endophyte *Delftia acidovorans* biotransformed polyphyllin VII (PPL7) to generate a new compound that overcame chemoresistance in breast cancer cells.

## RESULTS AND DISCUSSION

Extracts of *P. polyphylla* have been used to treat liver and lung cancer as well as laryngeal carcinoma in traditional Chinese medicine [15]. Saponins have been identified as major bioactive constituents in *P. polyphylla* with anti-tumor activity [13;14;18]. To better understand the antitumor activity of these saponins, as well as develop more potent saponin-based agents, we used the endophyte *Delftia acidovorans* to biotransform polyphyllin and analyzed the pharmacological activity of the transformed products on breast cancer cells.

Since the type of endophytes in plants differs in differing environments, we collected both artificially-planted and wild *P. polyphylla* to find candidate endophytes. Because artificial planting allows plants to grow in more fertile soil with less threat from insects and parasites, they may contain higher levels of active components. On the other hand, endophytes from wild *P. polyphylla* face a harsher and more complex environment, which some species of endophyte may have developed diverse mechanisms to cope with. Initially, we isolated 44 endophytes from separate colonies formed by wild, and 40 endophytes from artificially-planted *P. polyphylla*. The percentages of spore-forming and Gram-positive and -negative bacteria were similar in the wild and artificially-planted tubers; more than half of the endophytes were Gram-negative (Table S1).

To identify their genera, the 16S rDNA of isolated endophytes was amplified by PCR, sequenced, and blasted against the bacterial genomes in the NCBI GenBank (Table S2a-c). The endophytes from wild *P. polyphylla* belonged to 8 genera: *Bacillus*, *Enterobacter*, *Klebsiella*, *Serratia*, *Pseudomonas*, *Stenotrophomonas*, *Pantoea*, and *Delftia*. *Bacillus* was the most frequently found. On the other hand, the endophytes from artificially-planted *P. polyphylla* belonged to 6 genera: *Bacillus*, *Enterobacter*, *Klebsiella*, *Serratia*, *Pantoea*, and *Xanthomonas*. *Bacillus* was also the most frequently found genus. Therefore, these data suggest that wild *P. polyphylla* host more genera of endophytes*,* and the genera differ in plants from different sources. The similarities suggest that some endophytes are common to plants from different sources, but this needs further testing.

**Figure 1:**
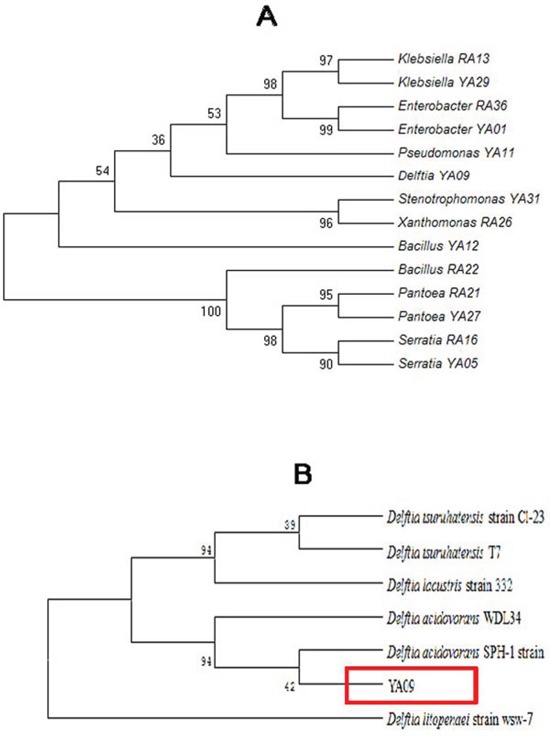
Phylogenetic analysis of endophytic bacteria **A.** Phylogenetic analysis of 16S rDNA from endophytic bacteria whose genera were found in both wild and artificially-planted *P. polyphylla.* The phylogeny was analyzed using MAGA4. Values near the branches are percentages of replicate trees with 500-times bootstraps. **B.** Phylogenetic tree constructed between known species of *Delftia* from the NCBI database and YA09 endophytes found in this study.

We then used the neighbor-joining method in MAGA4 software to construct the phylogenetic trees of endophytes whose genera were found in both wild and artificially-planted *P. polyphylla*. The 16S rDNA of *Klebsiella* (RA13 and YA29), *Enterobacter* (RA36 and YA01), *Pantoea* (RA21 and YA27), and *Serrati (*RA16 and YA05) showed high similarity in the wild and artificially-planted plants (Figure 1A).

Furthermore, another phylogenetic tree was constructed between the known species of *Delftia* in the NCBI database and the YA09 endophytes found in this study (Figure 1B). YA09 belongs to the *Delftia* genus and showed biotransformation activity (see below). To date, only 4 species of *Delftia* have been found: *D. tsuruhatensis*, *D. lacustris*, *D. acidovorans*, and *D. litopenaei*. YA09 fell into the same taxon as *D. acidovorans* SPH-1 strain.

**Figure 2:**
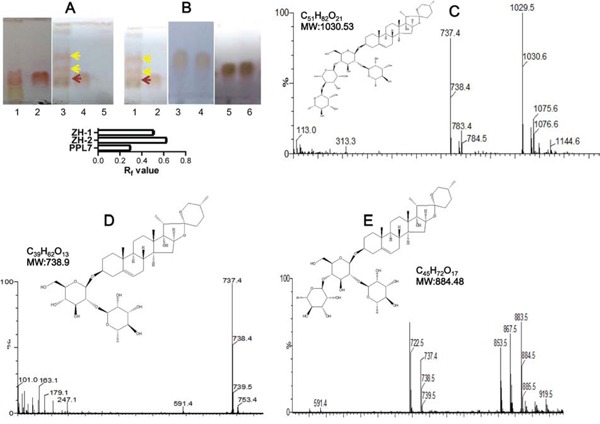
Biotransformation of PPL7 by endophyte YA09 (*Delftia acidovorans*) **(A)** PPL7 was biotransformed into two new compounds during culture with YA09. The culture medium after biotransformation at 30°C for 5 days was spotted on a TLC plate and separated by solvent mixture (chloroform: methanol = 3:1). Lane 1: culture medium with PPL7, without YA09 but incubated at 30°C for 5 days; lane 2: reference material of PPL7; 3 μg/ml PPL7 was added to the culture medium; lane 3: culture medium after biotransformation of PPL7 by YA09. Yellow arrows, new compounds (lower, ZH-1; upper, ZH-2); red arrow, PPL7; lane 4: culture medium with YA09 and PPL7 before biotransformation; lane 5: culture medium with YA09 and without PPL7. Histogram: statistical analysis of R_f_ values for PPL7, ZH-,1 and ZH-2 in (A) and (B). **(B)** Products of biotransformation were separated by column chromatography to obtain ZH-1 and ZH-2. Lane 1: culture medium after biotransformation of PPL7 by YA09; lane 2: culture medium before biotransformation; lanes 3 and 4: chromatography-purified ZH-1; lanes 5 and 6: chromatography-purified ZH-2. **(C-E)** ESI-MS data and chemical structures of PPL7 (C), ZH-1 (D), and ZH-2 (E).

We next examined the biotransformation activity of 84 endophytes on polyphyllins including polyphyllin I, II, V and VII (PPL7). Different polyphyllins were added to the culture system of each endophyte. After appropriate incubation, the biotransformation products in the culture medium were separated and visualized by thin-layer chromatography (TLC). Some of the incubations showed possible biotransformation because new spots in addition to the substrate polyphyllin appeared on the TLC. While in others, no new spots were found (Figure S1). The results showed marked evidence of biotransformation of PPL7 by endophyte YA09. Two new spots were found on the TLC plate, and their retardation factor (R_f_) values differed from that of PPL7 (Figure 2A), so we conclude that YA09 biotransformed PPL7 to produce two new compounds that we named ZH-1 and ZH-2. PPL7 was stable in the culture medium (Figure 2A lane 2), even when incubated at 30°C for 5 days (Figure 2A lane 1), and YA09 alone did not produce any new saponin-like compound (Figure 2A lane 5). Therefore, the new compounds must have been generated by YA09 metabolism of PPL7. Since they had the same color as the reference PPL7 sample in the TLC system, their chemical structure most likely belonged to the saponins.

We then separated the two compounds by column chromatography into ZH-1 and ZH-2 (Figs S2 and 2B) and their chemical structures were analyzed and compared with PPL7. Because few previous studies have reported the precise structure of PPL7 and these studies differ [19–21], the chemical structure of PPL7 was analyzed first by electrospray ionization–mass spectrometry (ESI-MS), ^1^H nuclear magnetic resonance (NMR), and ^13^C NMR. The ESI-MS data showed that m/z = 1029.5 (t = 3.80 min) was the standard molecular ion ([M-H]-), and loss of the 1 and 2 rhamnose groups led to daughter ions with peaks at m/z = 883.5 and 737.4, respectively (Figure 2C). ^1^H NMR (pyridine-d5, 400 MHz) showed different types of H-atom signals: δ: 0.696 (^3^H, d, J = 5.6 Hz, H-27), δ: 0.979 (^3^H, s, H-1), δ: 1.104 (^3^H, d, H-19), δ: 1.238 (^3^H, d, J = 7.16 Hz, H-21), δ: 1.780 (^3^H, d, J = 6.0 Hz, rhaH-61), δ: 1.620 (^3^H, d, J = 6.0 Hz, rhaH-62), δ: 1.620 (^3^H, d, J = 6.0 Hz, rhaH-63), δ: 3.516 (^2^H, m, H-26), δ: 3.879 (^1^H, m, H-3), δ: 4.947 (^1^H, d, J = 4.2 Hz, gluH-1), δ: 5.861 (^1^H, m, rhaH-12), δ: 6.308 (^1^H, m, rha H-13), and δ: 6.423 (^1^H, s, rhaH-11). The results of ^13^C-NMR (pyridine-d5, 100 MHz, Table S3) revealed that PD2 exhibits a C=C bond C-atom with signals at δ 141.24 (C-5) and δ 122.30 (C-6); four anomeric C-atoms with signals at δ 100.78 (gluC-1), δ 102.65 (rhaC-11), δ 102.69 (rhaC-12), and δ 103.78 (rhaC-13); and seven CH_3_ C-atom signals at δ 17.77 (C-27), δ 17.61 (C-18), δ 19.91 (C-19), δ 10.23 (C-21), δ 18.90 (rhaC-61), δ 19.35 (rhaC-62), and δ 19.21 (rhaC-63) were also detected. Therefore, the structure of PPL7 is pennogenin-3-O-α-L-rhamnopyranosyl(1→4)-α-L-rhamnopyranosyl(1→4)-[α-L-rhamnopyranosyl(1→2)]-β-D-glucopyranoside (Figure 2C), which is consistent with the study of Wang et al [21].

Then, ZH-1 was analyzed using the same methods. The ESI-MS data showed that m/z = 737.4 (t = 5.02 min) was the standard molecular ion ([M-H]-) (Figure 2D). Loss of a rhamnose group led to a daughter ion with a peak at m/z = 591.4. ^1^H NMR (pyridine-d5, 400 MHz) showed different types of H-atom signals: δ: 0.706 (^3^H, d, J = 5.6 Hz, H-27), δ: 0.979 (^3^H, s, H-18), δ: 1.104 (^3^H, d, H-19), δ: 1.238 (^3^H, d, J = 7.16 Hz, H-21), δ: 1.780 (^3^H, d, J = 6.0 Hz, rhaH-61), δ: 3.516 (^2^H, m, H-26), δ: 3.879 (^1^H, m, H-3), δ: 4.947 (^1^H, d, J = 4.2 Hz, gluH-1), and δ: 6.393(^1^H, s, rhaH-11). The 13C-NMR results (Table S3) exhibited a C=C bond C-atom with signals at δ 141.24 (C-5) and δ 122.27 (C-6); two anomeric C-atoms with signals at δ 100.72 (gluC-1) and δ 102.40 (rhaC-11); and five CH_3_ C-atom signals at δ 17.79 (C-27), δ 17.80 (C-18), δ 19.87 (C-19), δ 10.71 (C-21), and δ 19.13 (rhaC-61) were also detected. Therefore, the structure of ZH-1 is pennogenin-3-O-α-L-rhamnopyranosyl(1→2)-β-D-glucopyranoside, which is equivalent to polyphyllin VI (Figure 2D) [21].

Finally, ZH-2 was analyzed. The ESI-MS data showed that m/z = 883.5 (t = 4.80 min) was the standard molecular ion ([M-H]-) (Figure 2E). Loss of one and two rhamnose groups led to daughter ions with peaks at m/z = 737.4 and 591.4, respectively. ^1^H NMR (pyridine-d5, 400 MHz) showed: δ: 0.696 (^3^H, d, J = 5.6 Hz, H-27), δ: 0.979 (^3^H, s, H-1), δ: 1.104 (^3^H, d, H-19), δ: 1.238 (^3^H, d, J = 7.16 Hz, H-21), δ: 1.780 (^3^H, d, J = 6.0 Hz, rhaH-61), δ: 1.620 (^3^H, d, J = 6.0 Hz, rhaH-62), δ: 3.516 (^2^H, m, H-26), δ: 3.879 (^1^H, m, H-3), δ: 4.947 (^1^H, d, J = 4.2 Hz, gluH-1), δ: 5.802 (^1^H, m, rhaH-12), and δ: 6.423 (^1^H, s, rhaH-11). Furthermore, 13C-NMR (Table S3) exhibited a C=C bond C-atom with signals at δ 141.24 (C-5) and δ 122.30 (C-6); three anomeric C-atoms with signals at δ 100.75 (gluC-1), δ 102.58 (rhaC-11), and δ 102.60 (rhaC-12); and six CH_3_ C-atom signals at δ 17.77 (C-27), δ 17.73 (C-18), δ 19.89 (C-19), δ 10.67 (C-21), δ 19.02 (rhaC-61), and δ 19.68 (rhaC-62) were also detected (Table S3). As a result, the structure of ZH-2 is pennogenin-3-O-α-L-rhamnopyranosyl(1→4)-[α-L-rhamnopyranosyl(1→2)]-β-D-glucopyranoside (Figure 2E). We searched previous studies, and conclude that ZH-2 is a novel chemical.

In the transformation of PPL7 by *D. acidovorans*, the loss of one and two rhamnose groups led to the formation of ZH-1 and ZH-2, suggesting that the main enzyme involved is a rhamnosidase. However, the efficacy of the rhamnosidase in *D. acidovorans* was only ~15% during the transformation of PPL7 (50 mg PPL7 yielded ~7.5 mg ZH-2). Therefore, bioengineering methods are needed to enhance the activity of rhamnosidase in *D. acidovorans* if further studies confirm that ZH-2 is a good anti-chemoresistance agent in cancer. For example, the production of rhamnosidase in *D. acidovorans* could be boosted by inserting a strong promoter for the rhamnosidase gene. Furthermore, rhamnosidase exists in many plants, yeasts, fungi, and bacteria, so other sources could be used to develop ZH-2. On the other hand, rhamnosidase is an important enzyme in a variety of processes in the food, wine, and chemical manufacturing industries [22]. While most α-rhamnosidases are active on the α-1, 2 glucosidic linkage [22], the rhamnosidase in *D. acidovorans* tends to act on the α-1, 4 glucosidic linkage, so this endophyte provides a new source of rhamnosidases with unusual substrate specificity.

**Figure 3:**
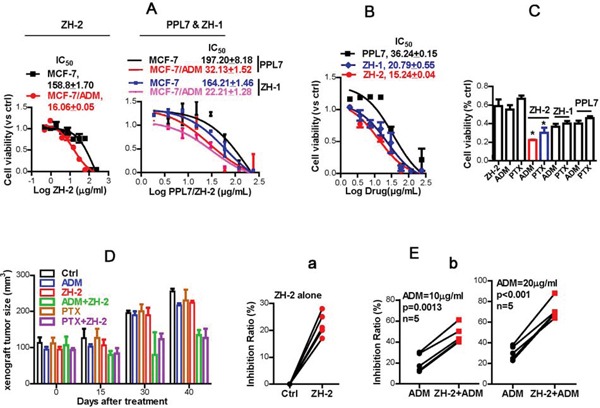
ZH-2 decreases chemoresistance in breast cancer cells **A.** ZH-2, ZH-1, and PPL7 killed more chemoresistant MCF-7/ADM cells than chemosensitive MCF-7 cells. Cytotoxicity was calculated from MTT assays; the IC_50_ (μg/ml) was determined by nonlinear regression. **B.** ZH-2 inhibited the growth of MCF-7/ADM cells more than PPL7 and ZH-1. Cytotoxicity was determined by MTT. **C.** Combinations of ZH-2, ZH-1, or PPL7 (15 μg/ml) with ADM (10 μM) or PTX (10 μM) were able to inhibit the proliferation of MCF-7/ADM cells. The viability was analyzed using MTT. *p <0.05 *vs* ADM or PTX alone. **D.** Combinations of ZH-2 and ADM or PTX inhibited the growth of xenografts from MCF-7/ADM cells. The xenografts were allowed to grow up to ~100m^3^ after MCF-7/ADM inoculation (0 days after treatment), then the drugs (3 mg/kg i.p. one time every 3 d for ADM, PTX or ZH-2) were applied for 40 days to assess the volume change in xenografts. **E.** (a) ZH-2 (15 μg/ml) killed few primary human breast cancer cells that were resistant to ADM. (b) Combination of ZH-2 (15 μg/ml) with ADM (10 and 20 μg/ml) enhanced the anti-cancer effect of chemotherapy on chemoresistant primary breast cancer cells.

Part of our studies focus on the mechanism of chemoresistance in breast cancer, and polyphyllins show anti-breast cancer activity [13], so we studied the anti-chemoresistance effect of ZH-2 in breast cancer and compared it with PPL7 and ZH-1 (polyphyllin VI). Chemoresistant MCF-7/ADM cells and their parental control MCF-7 cells were used. The MCF-7/ADM cells were induced by long-term treatment with adriamycin (ADM). This cell line is more malignant than the parental control [23–25] and is resistant to both ADM and paclitaxel (PTX) [24;25]. We first determined whether PPL7 and its derivatives inhibit the growth of MCF-7/ADM cells. Interestingly, PPL7, ZH-1, and ZH-2 killed more MCF-7/ADM cells than MCF-7 cells (Figure 3A). Also, ZH-2 inhibited the growth of MCF-7/ADM cells more strongly than ZH-1 or PPL7 (Figure 3B).

The findings that PPL7 and its derivatives killed more chemoresistant MCF-7/ADM cells suggest that these agents may antagonize chemoresistance. We found that ZH-2 re-sensitized MCF-7/ADM cells, and improved the anti-cancer activity of commonly-used chemotherapeutic agents. Although ZH-2 (15 μg/ml) and ADM or PTX (10 μg/ml) at a dose near the IC_50_ only killed 50-60% of MCF-7/ADM cells, ~80% of these cells were killed when ZH-2 was combined with ADM or PTX, and the activity of ZH-2 was better than that of ZH-1 and PPL7 (Figure 3C). Therefore, these data suggest that PPL7 and its derivatives inhibit chemoresistance in breast cancer cells, and ZH-2 has the highest activity. The difference in activity may be due to the structural alteration of PPL7 and its derivatives. We found that the inhibitory effect of PPL7 and its derivatives on cell viability and chemoresistance increased with the loss of rhamnose groups. This may allow ZH-2 to enter cells and reach its target more easily, but this requires further investigation. Having obtained an active product, we next focused on the mechanism by which ZH-2 inhibited chemoresistance.

Consistent with the results of *in vitro* studies, chemoresistant xenografts from MCF-7/ADM cells were not readily diminished by ADM and PTX. However, ADM or PTX in combination with ZH-2 effectively decreased the growth of such xenografts (Figure 3D).

We then collected primary breast cancer cells from five patients, whose cancer cells showed ADM-resistance in clinical drug-sensitivity tests (<50% cells were killed by ADM). ZH-2 alone killed 22 ± 1.93% of these primary cancer cells (Figure 3Ea), and 10 and 20 μg/ml ADM killed 20.2 ± 3.9% and 30.4 ± 2.9%, respectively (Figure 3Eb). However, when ADM was combined with ZH-2, anti-cancer effect markedly increased to 47.0 ± 3.9% and 71.8 ± 4.2%, respectively (Figure 3Eb).

**Figure 4:**
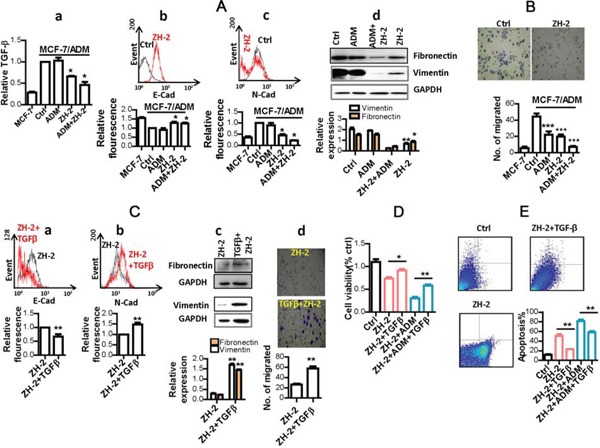
ZH-2 inhibited the EMT pathway in MCF-7/ADM cells **A.** MCF-7/ADM cells were treated with ZH-2 (15 μg/ml) and/or ADM (10 μM), then the hallmarks of EMT were analyzed in MCF-7/ADM and MCF-7 cells. (a) ELISA analysis of extracellular active TGF-β. (b) and (c) Flow cytometry of cell-surface E-cadherin (E-Cad) and N-cadherin (N-Cad). (a~c) The value for MCF-7/ADM cells without any treatment was set at 1. (d) Western blot analysis of vimentin and fibronectin expression. **p<0.01, *p<0.05 *vs* control. **B.** Cell migration with different treatments. ***p <0.0001 *vs* control.**C.** Treatment with TGF-β reversed the decrease in hallmarks of the EMT pathway. MCF-7/ADM cells were treated with ZH-2 (15 μg/ml) with or without TGF-β (5 ng/ml) for 48 h, then cell-surface E-Cad (a), cell-surface N-Cad (b); vimentin and fibronectin (c); and cell migration (d) were assessed. **p <0.01 *vs* control. (a, b) The value for MCF-7/ADM cells with ZH-2 treatment was set at 1 **D.** Treatment with TGF-β enhanced the viability of MCF-7/ADM cells under ZH-2 or ADM treatment as assessed by MTT. Cells were treated with ZH-2 (15 μg/ml) and/or ADM (10 μM) with or without TGF-β (5 ng/ml) for 48 h. *p <0.05, **p <0.01 *vs* without TGF-β. **E.** Treatment with TGF-β decreased the apoptosis due to ZH-2 as assessed using an AnnexinV-FITC/PI kit. MCF-7/ADM cells were treated with ZH-2 (15 μg/ml) and/or ADM (10 μM) with or without TGF-β (5 ng/ml) for 48 h. **p <0.01 *vs* without TGF-β.

ZH-2 selectively killed and re-sensitized malignant MCF-7/ADM cells suggest that it targets signaling pathways that are essential for both proliferation and drug sensitivity in malignant cancer cells. Previously, we showed that MCF-7/ADM cells display typical features of the epithelial-mesenchymal transition (EMT), such as high motility, high expression of vimentin, and cell-surface N-cadherin [23;26]. More importantly, others' and our study have shown that the EMT process is required by malignant cells to promote proliferation, and is also tightly associated with chemoresistance, so inhibiting the EMT process could effectively decrease the growth and chemoresistance of cancer cells like MCF-7/ADM cells [23;26]. The EMT is usually initiated by aberrant production of growth factors such as transforming growth factor-β (TGF-β) and fibroblast growth factor in epithelial cancer cells, then signaling molecules such as Ras, Wnt, and Notch are activated to pass the growth signals to transcription factors such as Snai1 and Twist1. Finally, the hallmarks of mesenchymal cells, including fibronectin, vimentin and N-cadherin are expressed. When the EMT occurs in epithelial cancer cells, they are usually resistant to apoptosis (and therefore display chemoresistance), as well as having the high motility needed for invasion and metastasis [26;27]. Therefore, to understand the mechanism of ZH-2 in inhibiting chemoresistance, the activity of the EMT pathway was analyzed after ZH-2 treatment. First, TGF-β was measured because it is one of the most common activators of the EMT pathway. Usually, TGF-β is secreted in an inactive form as it is packaged into the latent complex. Only when it is released from the latent complex, can the active form of TGF-β trigger its associated signaling pathways [28]. Therefore, supernatants of MCF-7 cells were collected, and an enzyme-linked immunosorbent assay (ELISA) that only recognizes the active form of TGF-β was used. The result showed that MCF-7/ADM cells activated significantly more TGF-β than MCF-7 cells, and the active TGF-β was significantly decreased by ZH-2 (Figure 4Aa). Second, markers of the EMT pathway were analyzed. MCF-7/ADM cells showed the EMT hallmarks as we had previously demonstrated [23]: decreased cell-surface E-cadherin, increased cell-surface N-cadherin, high expression of vimentin and fibronectin (Figure 4Ab-d), and high motility (Figure 4B). However, after ZH-2 treatment, N-cadherin, vimentin, and fibronectin expression and cell motility were significantly decreased, while E-cadherin was increased. Combination of ZH-2 and ADM also strongly reduced the EMT hallmarks, and these decreases were associated with ZH-2 but not ADM, since ADM alone did not significantly change the levels of TGF-β, N-cadherin, vimentin, and fibronectin. Without changes in these markers, the EMT pathway is not involved [29]. Furthermore, the stronger inhibitory effect on the EMT of ZH-2 plus ADM than ZH-2 alone may be because the greater cytotoxicity of ADM and ZH-2 together induces a stronger apoptotic response (see Figure 4E). Then the complicated cross-talk between apoptosis and the EMT pathway may reinforce the effect of ZH-2 [30;31]. Together, these data thus suggest that inhibition of the EMT pathway by ZH-2 makes MCF-7/ADM cells vulnerable to the toxicity of both ZH-2 and chemotherapeutic agents. On the other hand, in normal MCF-7 cells with a lower degree of malignancy, ZH-2 showed less activity because the EMT does not occur in these cells.

To further confirm that the EMT is involved in the mechanism of ZH-2, we were able to rescue ZH-2-treated-MCF-7/ADM cells with recombinant TGF-β protein (Abcam, USA). As a result of TGF-β treatment together with ZH-2, N-cadherin, vimentin, and fibronectin expression were regained and cell motility was increased, while E-cadherin expression was inhibited (Figure 4Ca-d). Although these results confirmed the involvement of the EMT pathway, we must point out that the activation/inhibition induced by ZH-2 throughout the EMT signaling pathway requires further study.

One of the reasons that the EMT contributes to chemoresistance is that it increases proliferation and inhibits apoptosis [26;27]. We therefore investigated the relationship between the mechanisms by which ZH-2 inhibits the EMT and interferes with proliferation and apoptosis. After treatment with ZH-2, the viability of MCF-7/ADM cells decreased (Figures 3B and 4D), suggesting a low proliferation rate. When the EMT pathway was restored by recombinant TGF-β, the ZH-2-treated cells regained a high proliferation rate (Figure 4D). Furthermore, ZH-2 treatment induced high rate of apoptosis in MCF-7/ADM cells (Figure 4E), while such apoptosis was diminished by recombinant TGF-β and restoration of the EMT pathway. Therefore, these data indicate that ZH-2 blocks the high proliferation rate and insensitivity to apoptosis in chemoresistant breast cancer cells *via* the EMT pathway, so that the chemoresistance is inhibited.

Taken together, our results indicate that ZH-2 is a good candidate for the further development of anti-cancer drugs and anti-chemoresistance agents.

## MATERIALS AND METHODS

### Isolation of endophytes

Root tubers of wild and artificially-planted *P. polyphylla* were collected in Wenshan, Yunnan Province, P. R. China, and endophytes were isolated according to a previous protocol [11]. Briefly, the tuber was sterilized and ground. Potential endophytes were cultured on nutrient broth medium (NB; beef extract 5.0 g L^−1^, peptone 10 g L^−1^, and NaCl 5.0 g L^−1^, pH 7.2-7.4) at 30°C until separate colonies were formed. The endophytes were preliminarily identified by spores and Gram-staining.

### Identification of endophytes by 16S ribosomal DNA sequencing

Genomic DNA was extracted from isolated endophytes using the chloroform/isoamyl alcohol-based method (24:1). The bacteria-specific primer 8F (5′AGAGTTTGATCCTGGCTCAG3′) and the universal primer 1492R (5′GGTTACCTTGTTACGACTT3′) were then used to amplify 16S rDNA by PCR at 95°C for 5 min; 30 cycles at 94°C for 30 s, 50°C for 40 s, and 72°C for 1.5 min; then 72°C for 10 min. The PCR products were then cloned into a plasmid pMDT-18 vector (Takara, Japan), transfected into *E. coli* JM109 strain, and cultured on Luria-Bertani medium containing ampicillin.

Transformants were selected and 16S rDNA was amplified with the universal primer M13 (forward: 5′CGCCAGGGTTTTCCCAGTCACGAC3′; reverse: 5′AGCGGATAACAATTTCACACAGGA3′) by PCR at 95°C for 5 min; 30 cycles at 94°C for 30 s, 50°C for 40 s, and 72°C for 1.5 min; then 72°C for 10 min. The DNA sequence of 16S rDNA was then analyzed by Sangon Biotech (Shanghai, China).

The resulting sequences were analyzed with the BLAST algorithm of NCBI. Phylogenetic trees were constructed using MAGA4 software.

### Biotransformation of saponins of *Paris polyphylla* by endophytes

The endophytes were cultured in 5 ml NB medium at 30°C to reach the logarithmic phase, then 0.2 mg/ml polyphyllin I, II, V, or PPL7 was added. The endophytes were cultured at 30°C for a further 5 days, then the medium was centrifuged and the supernatant extracted with butyl alcohol for 8 h. The product of extraction was distilled into a yellow powder which was dissolved in 0.5 ml methanol for thin-layer chromatography (TLC). To perform biotransformation for column chromatography analysis, the reaction was amplified in a 50-ml NB system.

### TLC analysis of the products of biotransformation

TLC is a chromatography technique to separate and detect different nonvolatile compounds in a mixture [32]. To perform TLC, the bottom of the 20 × 20 cm TLC plate was placed in a chamber filled with the solvent mixture (chloroform: methanol = 3:1). Then the samples of biotransformation were spotted on the plate above the surface of the solvent. Due to differences in solubility in the solvent, different compounds in the samples were then drawn up with different rate of ascent by organic solvent via capillary action, so they were separated and shown as dissociative spots on the plate. After capillary action, the separated spots were stained with 5% sulfuric acid in ethanol. The rate of ascent of each spot was quantified by the retardation factor (R_f_), which was calculated as: R_f_ = migration distance of spot / migration distance of solvent front.

### Separation of saponin monomers in the products of biotransformation

The products of biotransformation dissolved in methanol were isolated by column chromatography (silica gel; diameter:height = 1:50) eluted with chloroform:methanol (200:1, 100:1, 50:1, and 20:1). One fraction (fA) contained saponins, as identified by TLC. This fraction was isolated by column chromatography [silica gel (Qingdao Haiyang Chemical, China); diameter:height = 1:80] eluted with chloroform:methanol (50:1) to generate fA1 and fA2. These fractions were purified again by column chromatography (Sephadex LH-20; Amersham Pharmacia, Sweden); diameter:height = 1:80) eluted with methanol. The products were dried into ZH-1 and ZH-2. The protocol for column chromatography is shown in Figure S2.

### Identification of the structures of products of biotransformation

Electrospray ionization–mass spectrometry (ESI-MS, Waters, USA), proton nuclear magnetic resonance (^1^H NMR) and carbon-13 nuclear magnetic resonance (^13^C NMR) (Bruker Avance III Spectrometer, Switzerland) were used to identify the structures of ZH-1 and ZH-2.

### Cell cultures and treatments

Human breast cancer MCF-7 cells were from the ATCC (USA). Adriamycin (ADM)-resistant cells (MCF-7/ADM cells) were derived as previously described [24] by treating MCF-7 cells with stepwise increasing concentrations of ADM for 8 months. All cells were cultured in RPMI with 10% fetal bovine serum (FBS) at 37°C in a humidified atmosphere with 5% CO_2_.

For the treatment of MCF-7 cells with PPL7, ZH-1, or ZH-2, 10^5^ cells were seeded into each well of 96-well plates and cultured overnight. Then the cells were treated with different concentrations of PPL7, ZH-1, or ZH-2 (0.97–250 μg/mL, obtained through two-fold serial dilutions) for 48 h in serum-free medium. Then the cytotoxicity of the drugs was analyzed by MTT assay (Invitrogen, USA), and the IC_50_ was calculated by nonlinear regression in GraphPad prism software. For experiments using combinations of ZH-2 with ADM or paclitaxel (PTX) or the EMT activator transforming growth factor-β (TGF-β, Abcam, USA), 15 μg/ml ZH-2 and 10 μM ADM or PTX, or 10 ng/ml TGF-β were used to treat MCF-7/ADM cells simultaneously for 48 h, then the cell viability was analyzed by MTT and compared with Student's t-test or one-way ANOVA in GraphPad prism software.

### Flow cytometry

The cell-surface expression of N-cadherin and E-cadherin was determined by flow cytometry [33]. Cells were incubated with anti-N-cadherin or anti-E-cadherin (Santa Cruz Biotechnology, USA) at 4°C for 30 min, washed, and then stained with secondary antibody with FITC for 30 min. The cells were then fixed in 0.5% formaldehyde. To analyze apoptosis, cells were stained with Annexin V-FITC/PI dye from an apoptosis detection kit (Beyotime Biotechnology, PR China). Fluorescence was detected by a BD FACScan flow cytometer (Becton Dickinson, USA).

### Enzyme-linked immunosorbent assay

The extracellular concentrations of TGF-β were evaluated using ELISA kits (R&D Systems Inc., USA). Briefly, supernatants of MCF-7/ADM or MCF-7 cells were coated onto 96-well plates, and biotinylated anti-human active TGF-β antibody was added. Horseradish peroxidase-conjugated streptavidin was used for color development with 3,3′ 2,5,5′ 2-tetramethylbenzidine substrate. The color intensity was measured at 450 nm.

### Western blot

Cells were lysed and equal amounts of total protein were resolved on SDS-PAGE gels, transferred onto polyvinylidene difluoride membranes, and incubated with vimentin or fibronectin antibody (Abcam, USA); anti-β-actin served as a loading control. After incubation with appropriate secondary antibodies, antigen-antibody interaction signals were recorded with a chemiluminescence detection system and quantified by ImageJ software (NIH, Bethesda, MD).

### Cell migration assay

Cell migration assays were performed following a previous protocol [23]. Briefly, 10^5^ cells were added to the top chamber of 24-well Corning plates with uncoated PET membranes. Medium with 10% FBS was added to the lower chamber as a chemoattractant. Cells were allowed to migrate through the PET membranes for 48 h. Then the cells on the upper surface of membrane that had not migrated were removed with a cotton swab. Cells that had migrated to the lower surface were fixed in 4% (m/v) paraformaldehyde, stained with crystal violet, and counted under a microscope.

### In vivo study

MCF-7/ADM cells were injected into the flanks of female nude mice (5 × 10^6^ cells per mouse) and tumor growth was recorded every 5 days using calipers until they reached ~100 mm^3^. Then the mice were injected with ADM or PTX (3 mg/kg i.p. once every 3 days) and/or ZH-2 (3 mg/kg i.p. once every 3 days) at the tumor sites (n = 5 for each treatment).

### Clinical drug-sensitivity test

Each patient involved in this study provided written informed consent. All studies enrolling patients or their sample analysis were conducted with approval from the Ethics Committees of the university and hospital. Primary breast cancer cells were freshly separated from biopsy specimens of diagnosed breast tumors. Viable cells were separated and cultured in DMEM/F 12 medium (Gibco, Canada) containing 10% fetal bovine serum (Gibco) and treated with ADM (10 or 20 μg/ml) and ZH-2 (15 μg/ml) simultaneously for 24 h. Cytotoxicity was analyzed using MTT assays.

## SUPPLEMENTARY FIGURES AND TABLES



## Abbreviations

PPL7: polyphyllin VII
TLC: thin-layer chromatography
ADM: adriamycin
PTX: paclitaxel
TGF-β: transforming growth factor-beta
EMT: epithelial-mesenchymal transition
ESI-MS: electrospray ionization–mass spectrometry
