# Supplementary material for: A new agent developed by biotransformation of polyphyllin VII inhibits chemoresistance in breast cancer
Source: Oncotarget. 2015 Dec 19;7(22):31814–24. doi: 10.18632/oncotarget.6674 (PMC5077978; doi:10.18632/oncotarget.6674)
Supplement: Supplementary file 1 [file oncotarget-07-31814-s001.pdf]

A new agent developed by biotransformation of polyphyllin VII inhibits chemoresistance in breast cancer

SUPPLEMENTARY FIGURES AND TABLES

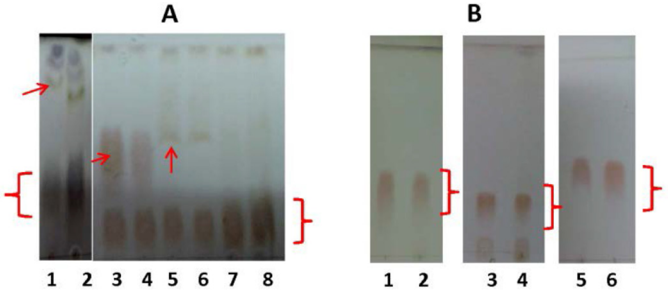

Supplementary Figure S1: Examples of biotransformation on polyphyllins by different endophytes. A. Examples of endophytes that show possible biotransformation activities on PPL7. PPL7 was biotransformed by endophytes YA11 (lane 1 and 2), YA30 (lane 3 and 4), RA06 (lane 5 and 6), RA01 (lane 7 and 8) at 30°C for 3~7 days (please refer table S2 for genera of these endophytes), the products were analyzed by TLC. Arrows indicate new compounds found in the culture medium after biotransformation, and brackets indicate bands for PPL7. YA11, YA30 and RA06 showed possible activity in biotransformation, while RA01 did not. **B.** YA09 did not show activity in biotransformation in other polypyllins. Effects of YA09 on polyphyllin I (lane 1 for control, lane 2 for transformed products), polyphyllin II (lane 3 for control, lane 4 for transformed products) and polyphyllin V (lane 5 for control, lane 6 for transformed products) were analyzed by TLC. Biotransformation was performed at 30°C for 5 days. Brackets indicate bands for polyphyllins.

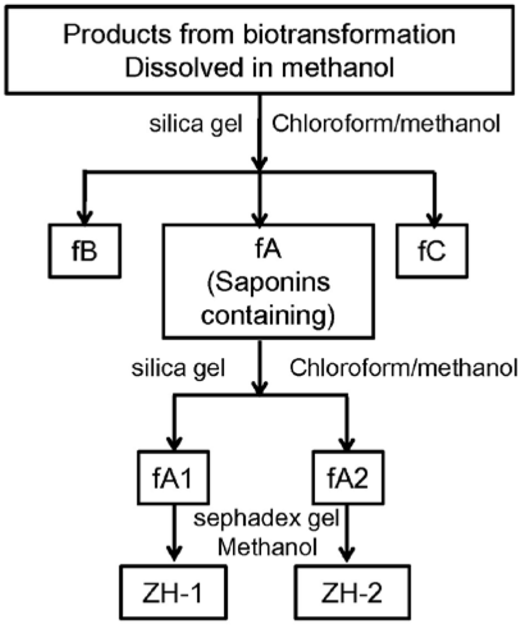

Supplementary Figure S2: Protocol for separation of the products from biotransformation. The culture medium from biotransformation was extracted in butyl alcohol, distilled, and dissolved in methanol, then isolated by silica gel column chromatography eluted with chloroform/methanol to generate fragment fA. fA was then isolated with silica gel column chromatography eluted with chloroform/methanol for the second round, separating it into fA1 and fA2. The fA1 and fA2 were purified again by Sephadex LH-20 column chromatography eluted with methanol. The products were finally dried into ZH-1 and ZH-2, respectively.

Supplementary Table S1: Types of endophytes in wild and artificially-planted *Paris polyphylla*

Wild: total 44			Artificially-planted: total 40		
**Spore-forming**	**Gram-positive**	**Gram-negative**	**Spore-forming**	**Gram-positive**	**Gram-negative**
16 (36.4%)	3 (6.8%)	28 (63.6%)	14 (35%)	3 (7.5%)	23 (57.5%)

Supplementary Table S2a: Summary of genera of endophytes from *Paris polyphylla*

	Genus	No. of strains	Percentage
Wild	*Bacillus*	16	36.4%
	Serratia	9	20.5%
	Klebsiella	6	13.6%
	Enterobacter	6	13.6%
	Pseudomonas	3	6.8%
	Stenotrophomonas	2	4.5%
	Pantoea	1	2.3%
	Delftia	1	2.3%
Artificially-planted	*Bacillus*	14	35%
	Enterobacter	11	27.5%
	Klebsiella	6	15%
	Serratia	5	12.5%
	Pantoea	3	7.5%
	Xanthomonas	1	2.5%

Supplementary Table S2b: Identification results of endophytic bacteria in wild Paris polyphylla,

ID	Closest relative	Accession	Identity(%)
YA01	*Enterobacter asburiae*	KC568141.1	99
YA02	*Enterobacter sp. JDG188*	JX035955.1	99
YA03	*Serratia sp. AS12*	CP002774.1	99
YA04	*Bacillus megaterium.PRE9*	EU880506.1	99
YA05	*Serratia plymuthica.4Rx13*	CP006250.1	99
YA06	*Klebsiella oxytoca.No.5*	AB353045.1	99
YA07	*Bacillus megaterium Bm-10*	GQ866974.1	99
YA08	*Bacillus sp. HNL20*	EU373361.1	99
YA09	*Delftia sp. hg-1*	KF059265.1	99
YA10	*Klebsiella sp. GX17*	HQ018868.1	99
YA11	*Pseudomonas corrugata. LMG 5036*	HE586389.1	99
YA12	*Bacillus cereus. HLSSD-5*	FJ749283.1	100
YA13	*Bacillus sp. S23440*	D84630.2	100
YA14	*Uncultured Klebsiella sp. JXS1-28*	JN873189.1	99
YA15	*Pseudomonas sp. BIHB 811*	DQ885950.1	99
YA16	*Bacillus toyonensis. BCT-7112*	CP006863.1	99
YA17	*Serratia ficaria.DSM 4569*	NR_041979.1	99
YA18	*Bacillus cereus. G8639*	AY138271.1	99
YA19	*Enterobacter sp. JDG188*	JX035955.1	99
YA20	*Bacillus megaterium. IHB B 1331*	GU186111.1	99
YA21	*Serratia plymuthica. AS9*	CP002773.1	99
YA22	*Serratia plymuthica. ATY36*	HQ219955.1	99
YA23	*Bacillus sp. I-102*	AB531391.1	99
YA24	*Serratia plymuthica.4Rx13*	CP006250.1	99
YA25	*Bacillus thuringiensis. CT-43*	CP001907.1	99
YA26	*Enterobacter sp. 2375*	JX174252.1	99
YA27	*Pantoea ananatis. AJ13355*	AP012032.1	99
YA28	*Bacillus cereuse*	AB334763.1	100
YA29	*Klebsiella sp. TNT*	DQ229100.1	99
YA30	*Bacillus cereus. 2000031486*	AY138272.1	99
YA31	*Stenotrophomonas maltophilia. Sea-8*	AF390080.1	99
YA32	*Serratia sp. R1(2008)*	EU236755.1	99
YA33	*Stenotrophomonas sp. ITCr01*	FR823396.1	99
YA34	*Enterobacter asburiae*	KC568141.1	99
YA35	*Enterobacter sp. B-14*	FJ190535.1	99

(*Continued*)

ID	Closest relative	Accession	Identity(%)
YA36	*Klebsiella sp. LB-2*	DQ831003.1	99
YA37	*Bacillus thuringiensis*	CP006863.1	100
YA38	*Serratia proteamaculans. DSM 4543*	NR_025341.1	99
YA39	*Pseudomonas fluorescens. MazP22*	JX885768.1	99
YA40	*Klebsiella sp. XW721*	EU545402.1	99
YA41	*Enterobacter sp. TMT1390*	AB308444.1	99
YA42	*Bacillus sp. J1*	FJ815201.1	99
YA43	*Serratia plymuthica.XB*	EF064206.1	99
YA44	*Bacillus cereus. PR15*	JQ435675.1	99

Supplementary Table S2c: Identification results of endophytic bacteria in artifically planted *Paris polyphylla*

ID	Closest relative	Accession	Identity(%)
RA01	*Bacillus thuringiensis .CCM15B*	CP006863.1	99
RA02	*Enterobacter sp.CTSP4*	EM855187.1	99
RA03	*Bacillus toyonensis. BCT-7112*	CP006863.1	99
RA04	*Pantoea agglomerans.*	KC153126.1	99
RA05	*Bacillus sp.h3(JX)*	AY862988.1	99
RA06	*Enterobacter asburiae .K03*	HM854374.1	99
RA07	*Serratia plymuthica. ATY36*	HQ219955.1	99
RA08	*Enterobacter sp. ALL-3*	KC211019.1	99
RA09	*Bacillus sp. SAP02_1*	JN872500.1	99
RA10	*Uncultured Klebsiella sp.*	JN873194.1	99
RA11	*Klebsiella sp. sctcc7*	HQ622335.1	99
RA12	*Bacillus megaterium. PRE9*	EU880506.1	99
RA13	*Klebsiella sp. TNT1*	DQ229100.1	99
RA14	*Bacillus sp. M07(2011)*	JN020667.1	99
RA15	*Serratia sp. AS12*	CP002774.1	99
RA16	*Serratia plymuthica.4Rx13*	CP006250.1	99
RA17	*Bacillus thuringiensis.CCM15B*	CP006863.1	99
RA18	*Enterobacter sp. db-3*	KF059262.1	99
RA19	*Enterobacter asburiae*	KC568141.1	99
RA20	*Bacillus sp. G11111*	AB531398.1	99
RA21	*Pantoea ananatis. BD 622*	DQ195523.1	99
RA22	*Bacillus cereus.JN267*	KF150501.1	99
RA23	*Pantoea sp. R22(2010)*	GU566350.1	99
RA24	*Bacillus cereus. XY2*	JQ435695.1	99
RA25	*Bacillus megaterium. Jz11*	JF833087.1	99
RA26	*Xanthomonas campestris*	CP000050.1	99
RA27	*Enterobacter aerogenes. LRC134*	JF772068.1	99
RA28	*Enterobacter sp. CTSP21*	EM855202.1	99
RA29	*Uncultured Klebsiella sp. JXS1-28*	JN873189.1	99
RA30	*Bacillus cereus. SEP-4*	KF228908.1	99
RA31	*Klebsiella sp. F51-1-2*	DQ277701.1	99
RA32	*Bacillus megaterium. PRE9*	EU880506.1	99
RA33	*Serratia plymuthica*	EM344964.1	99
RA34	*Enterobacter asburiae*	KC568141.1	99
RA35	*Bacillus sp. M07(2011)*	JN020667.1	99

(*Continued*)

ID	Closest relative	Accession	Identity(%)
RA36	*Enterobacter sp. B-14*	FJ190535.1	99
RA37	*Enterobacter cloacae*	AM778415.1	99
RA38	*Bacillus cereus. SEP-4*	KF228908.1	99
RA39	*Serratia plymuthica. RVH1*	AY394724.1	99
RA40	*Klebsiella sp. AN-4*	EU888474.1	99

Supplementary Table S3: The ^{13}C-NMR data of transformed products and PPL7

Position	PPL7	ZH-1	ZH-2
C-1	38.02	38.10	38.05
C-2	30.90	30.62	30.80
C-3	78.50	78.57	78.53
C-4	39.43	39.42	39.42
C-5	141.24	141.24	141.24
C-6	122.30	122.27	122.30
C-7	32.55	32.77	32.65
C-8	32.28	32.15	32.20
C-9	50.71	50.75	50.72
C-10	37.62	37.61	37.62
C-11	21.42	21.44	21.42
C-12	32.91	32.91	32.91
C-13	45.61	40.92	40.75
C-14	53.53	53.51	53.51
C-15	32.82	32.68	32.77
C-16	90.67	90.69	90.67
C-17	90.49	90.51	90.49
C-18	17.61	17.80	17.73
C-19	19.91	19.87	19.89
C-20	45.23	45.44	45.35
C-21	10.23	10.71	10.67
C-22	110.29	110.29	110.29
C-23	32.55	32.49	32.53
C-24	29.27	29.27	29.26
C-25	30.62	30.62	30.62
C-26	68.79	69.96	69.81
C-27	17.77	17.79	17.77
Sugar moiety			
	Glc	Glc	
C-1	100.78	100.72	100.78
C-2	78.41	78.96	78.55
C-3	77.47	77.89	77.67
C-4	78.17	72.20	74.16
C-5	78.20	77.46	78.03
C-6	61.66	62.96	62.08
	Rha1	Rha1	Rha1

(*Continued*)

Position	PPL7	ZH-1	ZH-2
C-1	102.65	102.40	102.58
C-2	74.49	73.27	73.88
C-3	72.98	72.95	72.95
C-4	80.88	74.62	79.16
C-5	67.16	67.33	67.13
C-6	18.90	19.13	19.02
	Rha2		Rha2
C-1	102.69		102.60
C-2	73.76		73.82
C-3	73.73		73.82
C-4	74.62		68.16
C-5	70.88		69.23
C-6	19.35		19.68
	Rha3		
C-1	103.78		
C-2	73.33		
C-3	73.12		
C-4	74.49		
C-5	70.02		
C-6	19.21		
